# Viruses and viral proteins 

**DOI:** 10.1107/S205225251402003X

**Published:** 2014-10-14

**Authors:** Nuria Verdaguer, Diego Ferrero, Mathur R. N. Murthy

**Affiliations:** a Institut de Biología Molecular de Barcelona, CSIC, Parc Científic de Barcelona, Baldiri i Reixac 15, 08028-Barcelona, Spain; b Molecular Biophysics Unit, Indian Institute of Science, Bangalore 560 012, India

**Keywords:** bacteriophages, genome delivery, fusion proteins, RNA-dependent RNA polymerases, viral proteases, viral receptors, viruses

## Abstract

For more than 30 years X-ray crystallography has been by far the most powerful approach for determining the structures of viruses and viral proteins at atomic resolution. The information provided by these structures, which covers many important aspects of the viral life cycle such as cell-receptor recognition, viral entry, nucleic acid transfer and genome replication, has extensively enriched our vision of the virus world. Many of the structures available correspond to potential targets for antiviral drugs against important human pathogens. This article provides an overview of the current knowledge of different structural aspects of the above-mentioned processes.

## Abstract 2

The X-ray structures of viruses and viral proteins currently available are providing high-resolution snapshots of viral molecular machineries, expanding our vision of the virus world and giving crucial information on potential targets for future antiviral therapies.

## 1. Introduction   

Viruses have evolved different strategies for their multiplication and propagation. The diversity and complexity of viral protein structures now available at near atomic resolution and stored in the Protein Data Bank (PDB; http://www.rcsb.org/pdb/) or in the specialized database Virus Particle Explorer (VIPER; http://viperdb.scrips.edu) (Carrillo-Tripp *et al.*, 2009 ▶) are the result of outstanding achievements of structural virologists. The PDB now treasures the three-dimensional structures of over 350 virus capsids, from more than 35 viral families and about 5000 protein structures coded by viral genomes. More than 90% of these structures have been determined by X-ray crystallography (PDB July 2014). These include not only structural proteins forming icosahedral capsids and other components of virus particles, such as proteins of cylindrical viruses or different components of tailed phages, but also many viral enzymes such as viral proteinases, and RNA and DNA polymerases.

Animal viruses may infect host cells by anchoring to an appropriate receptor molecule(s), which will trigger penetration of the entire virion or some of its components, always including the viral genome, into the cell. Unless the nucleic acid enters alone, viral genome replication and expression will require uncoating of the capsid and release of the internalized viral nucleic acid. Cell recognition, entry and uncoating frequently overlap and rely on quite different mechanisms depending on the virus species and host type.

In non-enveloped animal viruses, the mechanisms for genome delivery have not yet been characterized in detail, although a number of steps of the process have been described. Some well studied models include poliovirus (PV) and Human Rhinoviruses (HRV) both belonging to the *Picornaviridae* family that includes a large number of human and animal pathogens. In these viruses, uncoating can be mediated by receptor binding, by low pH, or by the cooperative effect of both factors, and appears to be linked to the inherent stability and dynamics of the capsid. The X-ray and cryo-electron micrography structures of different HRV2 uncoating intermediates revealed the structural alterations that take place in the virus architecture, allowing the delivery of the genomic RNA: the acidic pH induces the conversion of the native HRV2 virions into an expanded, porous uncoating intermediate called an A-particle. This conversion is accompanied by major changes in the RNA organization and interactions at the inner capsid wall that would facilitate the subsequent RNA egress through large capsid disruptions produced at the particle twofold symmetry axes (Pickl-Herk *et al.*, 2013 ▶; Garriga *et al.*, 2012 ▶). Similar uncoating mechanisms have also been described for PV (Bostina *et al.*, 2011 ▶).

Enveloped animal viruses also use a two-step process to release their genetic material into the cell: first they bind to specific cell-surface receptors anchored to the target cell membrane and then they induce fusion of the viral and cell membranes. Binding to the cell-surface receptor in these viruses is mediated by a viral glycoprotein embedded in the viral lipid envelope which specifically interacts with some cellular molecule, which typically is a membrane-associated glycoprotein, carbohydrate or glycolipid. Detailed knowledge of virus–receptor interactions is essential to understand different aspects that determine viral tropism, spread and pathogenesis. During the last few years, a large number of high-resolution structures of viral proteins in complex with their specific receptors have been reported, shedding light on these aspects. Very recently, Wang and co-workers characterized the interactions between the newly identified Middle East respiratory syndrome coronavirus and its target cell by solving the structure of the receptor-binding domain of the viral envelope spike glycoprotein bound to its cellular receptor, the dipeptidyl peptidase 4 (Wang *et al.*, 2013 ▶). The structural information obtained might serve as a guide for the development of therapeutics against this novel coronavirus.

Fusion of viral and cell membranes may occur either at the cell surface or after internalization of the virus particle. The process is driven by specialized viral glycoproteins, called fusogens, which are in metastable conformations in the virus particle. Once activated, these proteins initiate a series of conformational changes, favouring the fusion of the two membranes. At the end of the fusion process, the viral fusogens adopt highly stable conformations. The free energy liberated during the transition from the metastable pre-fusion to the highly stable post-fusion conformation drives the fusion process. Remarkably, the virus–cell membrane-fusion process appears to follow essentially the same intermediate steps as in other membrane fusions that occur for instance in vesicular fusion or in cell–cell fusion. Recent data from Felix Rey and colleagues (Pérez-Vargas *et al.*, 2014 ▶) unveil a striking structural relationship between the *Caenorhabditis elegans* cellular fusion protein EFF-1, believed to be involved in the development of multicellular organisms, and the class II viral fusion proteins, indicating the importance of an intricate exchange of genetic information between viruses and cells during the evolution of multicellular organisms.

Like animal viruses, bacteriophages also recognize receptors in the host-cell surface. However, because of the thickness and hardness of bacterial cell walls, the phage particles cannot enter the cells by endocytosis. Phages have evolved various mechanisms to transport their genomes across the bacterial cell wall. Tailed phages use a tail that penetrates the cell wall in a way similar to that of the needle of a hypodermic syringe. The nucleic acid genome is then injected into the cell, driven in part by the internal pressure built in the phage head during packaging of the nucleic acid. In contrast, tail-less phages rely on host organelles for genome transfer. Recent data from Michael Rossmann and colleagues (Sun *et al.*, 2014 ▶) demonstrate a novel mechanism of DNA delivery adopted by the tail-less coliphage ϕX174 that requires the tubular structure of DNA pilot protein H to be wide enough to allow the passage of two antiparallel strands of ssDNA: this tube acts as a tail for the translocation of the viral genome, but it protrudes from the virion only at the time of infection.

Plant viruses do not enter their hosts *via* receptor-mediated endocytosis because of the cell-wall barrier. They gain entry into cells by mechanical injury caused by the vectors that transmit them or by mechanical inoculation. From the primary site of infection, they move from cell to cell and systemically infect plants using specialized proteins called movement proteins encoded by their genome which alter the plasmodesmata, the communication channels between cells. However, some of the plant viruses have been shown to enter and replicate within yeast cells and also enter animal cells. For example, cowpea mosaic virus (CPMV) appears to preferentially enter cancer cells by interacting with a 54 kDa cell-surface protein, vimentin (Koudelka *et al.*, 2009 ▶). CPMV conjugated with the anti-cancer drug doxorubicin showed effective toxicity towards HeLa cells (Albajali *et al.*, 2013 ▶). The feasibility of using fluorescent mCherry-potato virus X as a probe for optical imaging in human cancer cells has been demonstrated (Shukla *et al.*, 2013 ▶). Several plant viral coat protein genes, when expressed in a suitable expression system, assemble into virus-like particles (VLPs). VLPs could be engineered biologically or by chemical modifications so as to possess specific surface characteristics. VLPs are increasingly being investigated for several applications because of their nanometre size range, symmetry, polyvalency, monodispersity, efficient and inexpensive production, biocompatibility and biodegradability (reviewed in Steinmetz, 2010 ▶). Studies performed on mice using CPMV and cowpea chrorotic mottle virus (CCMV) showed that both viruses entered different tissues. CPMV particles largely accumulated in the liver and spleen while CCMV was mostly found in the thyroid gland (Steinmetz, 2010 ▶).

RNA viruses typically encode their own RNA-dependent RNA polymerase (RdRP) to ensure genome replication within the infected cell. These enzymes are major targets for the development of antiviral compounds against the corresponding pathogens. A number of studies have revealed the structures of different RdRPs and provided a mechanistic insight to the RNA-synthesis process in the case of single-stranded positive-sense RNA viruses and double-stranded RNA viruses. However, such information is still lacking for single-stranded negative-polarity RNA viruses. The most complete sets of structural data are from Picornaviruses and Caliciviruses. These structures provide high-resolution snapshots of the conformations adopted by the enzyme in the different steps of the catalytic cycle. Ng and colleagues provide structural and biochemical evidence in Norovirus for a range of conformational states of the polymerase associated with RNA-template–primer binding, NTP binding, catalysis, RNA translocation and pyrophosphate release (Ng *et al.*, 2008 ▶). Such structural information is essential for the design of new antiviral therapies.

## 2. Virus–receptor interactions   

The initial event in the life cycle of a virus is its contact with the target cell. This encounter is mediated by interactions between components on the viral surface (capsid proteins or viral membrane anchored proteins) and several elements on the cellular membrane that can get attached to the virus. Most viruses establish contacts with cellular receptors which could be sialic acid bound molecules or proteins on the cell surface. Frequently, the first virus–cell contact is mediated by adhesion receptors that allow reversible attachment of the virus to particular cells or organs, mainly by means of electrostatic interactions. This initial interaction could lack specificity and is employed to enhance the encounter between virus and entry receptors, which irreversibly bind the virus, allowing them access to the intracellular milieu either by direct endocytosis/pinocytosis or by inducing fusion or penetration of the viral genome into the cell. 

**Figure 1:**
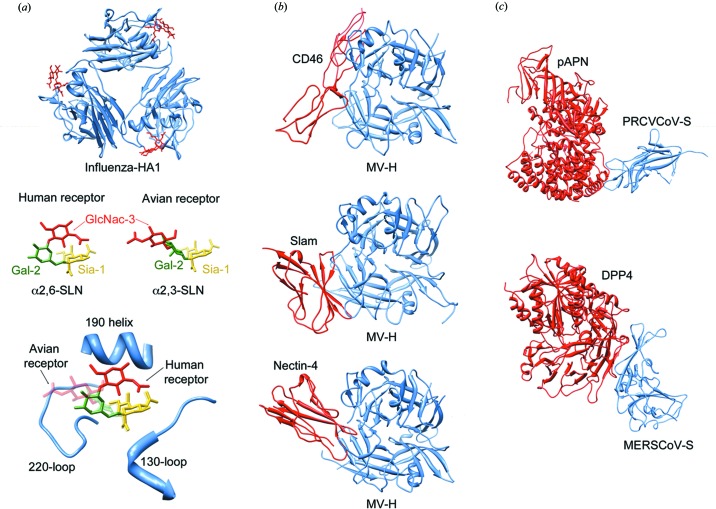
(*a*) The sialic acid binding pocket of influenza HA. A ribbon representation of an HA1 trimer is shown in the upper part. The structure of the bound human receptor analogue is shown as red sticks (PDB entry 4bh3, Xiong *et al.*, 2013 ▶). The components of the human (left) and avian (right) receptor analogues are shown in the middle part of the figure and a close-up of the receptor-binding pocket with the superimposition of the two ligands is shown in the lower part. (*b*) The MV-H protein bound to the receptors: CD46 (PDB entry 3inb, Santiago *et al.*, 2010 ▶; top), Slam (PDB entry 3alz, Hashiguchi *et al.*, 2011 ▶; middle) and nectin-4 (PDB entry 4gjt, Zhang *et al.*, 2013 ▶; bottom). Ribbon drawings, showing the MV-H molecules in blue and the different receptor molecules depicted in red. (*c*) Structure of the PRCV CoV S-APN complex (PDB entry 4f5c, Reguera *et al.*, 2012 ▶). (*d*) Structure of the complex MERS-CoV RBD-DPP4 complex (PDB entry 4l72, Wang *et al.*, 2013 ▶).

Characterization of the virus–receptor interactions has been a focus of sustained interest and many outstanding contributions of high-resolution X-ray structures of viral proteins in complex with their specific receptors have been reported during the last few years. Haemagglutinin (HA)–receptor interaction was extensively studied in different influenza virus subtypes. Influenza HA is synthesized in the infected cell as a polypeptide precursor (HA0) of about 550 amino acids that is proteolytically cleaved to generate the HA1 (approximately the N-terminal two thirds) and HA2 (the C-terminal third) chains that remain covalently linked by a disulfide bond. At the newly created HA2 N-terminus there is a stretch of hydrophobic amino acids, called the fusion peptide, which is inserted into the membrane during fusion (see the next section). The overall structure of the influenza HA resembles an elongated spike sticking out of the membrane. The distal head, formed exclusively by HA1 sequences, bears the receptor-binding site, formed by a shallow pocket exposed on an outward-forming surface. Different residues within this pocket mediate viral attachment to the α2,3- or α2,6-linked sialic acid moieties in avian or human cellular surfaces, respectively, as observed by X-ray crystallography in different complexes with receptor ana­logues. The sialic acid binding pocket of HA1 is formed by a loop–helix–loop structure composed of the 130-loop, 190-helix and 220-loop (Fig. 1 ▶). 

A repertoire of crystal structures of HA–receptor complexes from different influenza outbreaks has recently been reported, providing a picture of the evolutionary path for the emergence of the H3N2 Influenza strain between 1968 and 2002 (Lin *et al.*, 2012 ▶). Furthermore, this research revealed a number of features that contribute to the changes in receptor affinity. In particular, high-resolution X-ray structures have shown how the point mutation Asp225 to Asn in the 220-loop diminishes the specific HA–receptor interactions, justifying how this fixed mutation decreases the human receptor binding in H3N2 viruses circulating since the last decade, thereby reducing the impact of this influenza subtype. Moreover, the structures of different HA–receptor complex analogues have revealed the key determinants for animal-to-human transmissibility of the highly pathogenic avian H5N1: a single substitution (Gln to Leu) promotes binding to the human receptor to the detriment of the avian receptor (Xiong *et al.*, 2013 ▶). Interestingly, this mutation leading to increased hydrophobicity was responsible for the high transmissibility of human pandemic H2 and H3 influenza subtypes.

Recent crystallographic studies also revealed details of virus–host interactions for Measles virus (MV), which is a single-stranded, negative-sense, enveloped RNA virus. Three seminal works provide a landscape of atomic interactions between MV haemagglutinin (MV-H) and the protein receptors. The structure of MV-H bound to human CD46 fragment (Santiago *et al.*, 2010 ▶) provided a rationale for the critical role of only two residues (Tyr481 and Gly546) in the alternative use of CD46 as a virus receptor, preserving the affinity for the primary SLAM receptor. The structure of the complex between MV-H and SLAM has also been determined (Hashiguchi *et al.*, 2011 ▶). In addition, the structure of MV-H bound to a third receptor protein, nectin-4 which has been identified recently, has also been reported (Zhang, Lu *et al.*, 2013 ▶), showing that nectin-4 binds to MV-H by means of its N-terminal domain, establishing extensive hydrophobic inter­actions in the same concave lateral groove of MV-H as the other receptors but with minor differences (Fig. 1 ▶). These high-resolution structures that illustrate the variability of interactions important for MV infection can also explain the extended virus tropism mediated by the alternative use of receptors or the receptor-specificity switching. Additionally, this structural information provides a framework for rational antiviral design.

Other crystallographic data have allowed characterization of the early steps of infection in important pathogens such as the single-stranded, positive-sense RNA Coronaviruses (CoV). The spike glycoprotein (S) of the CoV particle forms characteristic surface projections that are employed to interact with the target cell receptors. The determinants of CoV tropism are located in the distal globular domain of this viral membrane glycoprotein, which mediates attachment of the virus to the cell-surface molecules. In fact, the structure of the porcine respiratory coronavirus (PRCV) receptor-binding domain of the S protein in complex with its cell-receptor protein, the aminopeptidase N (pAPN), shows that the conformation of the receptor-binding edge in the envelope S protein contains the determinants of their receptor-binding specificity (Fig. 1 ▶). This work also reports the interaction of a single glycan involved in protein interactions that must be conserved among a group of CoVs (Reguera *et al.*, 2012 ▶).

Very recently, Wang *et al.* (2013 ▶) determined the structure of the receptor-binding domain (RBD) present in the S protein of the newly identified Middle East respiratory syndrome coronavirus (MERS-CoV) in complex with its cellular receptor, the dipeptidyl peptidase 4 (DPP4). Interestingly, this receptor does not resemble any other CoV receptor in either sequence or structure. On the other hand, the MERS-CoV RBD appears to be different from the SARS-CoV reported earlier (Li *et al.*, 2005 ▶), despite the relative similarity in the core subdomain, contributing to the variance in receptor specificities (Fig. 1 ▶). The interphases of inter­actions consist of two regions distant from the DPP4 active site, where Li *et al.* (2005) identified relevant residues that support the viral–receptor interaction that will potentially guide therapeutic strategies against this virus.

## 3. Viral and eukaryotic fusion proteins   

The viral fusion glycoproteins structurally characterized to date fall into three classes, although the membrane-fusion pathway seems to be very similar for all the enveloped viruses studied so far.

**Figure 2:**
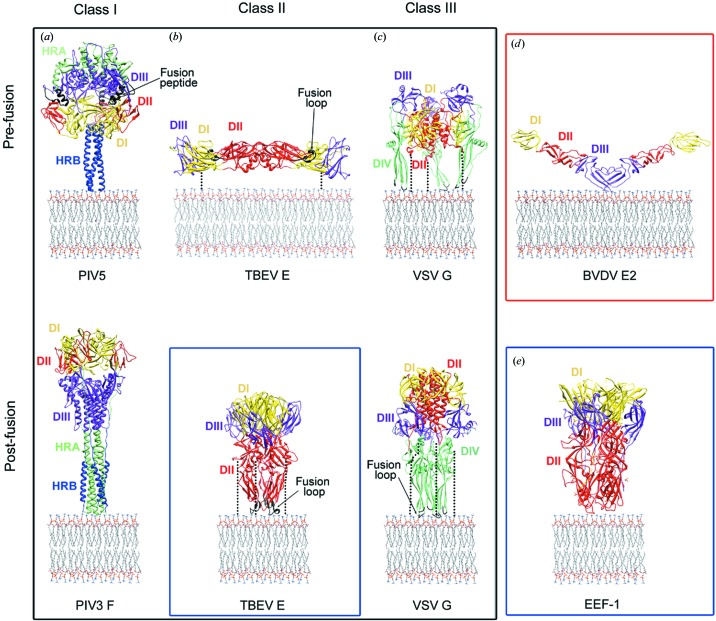
The three classes of viral fusion glycoproteins. Ribbon diagrams of the pre-fusion (top) and post-fusion (bottom) conformations of the paramyxovirus (class I; PDB entries 2b9b, Yin *et al.*, 2006 ▶, and 1ztm, Yin *et al.*, 2005 ▶) (*a*); flavivirus (class II; PDB entries 1svb, Rey *et al.*, 1995 ▶, and 1urz, Bressanelli *et al.*, 2004 ▶) (*b*); and rhabdovirus (class III; PDB entries 2j6j, Roche *et al.*, 2007 ▶, and 2cmz, Cho *et al.*, unpublished work) (*c*) proteins. For each class of fusogen, the equivalent protein domains are highlighted with identical colours and explicitly labelled (DI, yellow; DII, red; DIII, purple; DIV and heptad repeat region A, HRA, light green; HRB, blue; fusion peptide or loops, black). (*d*) The novel fold of the envelope protein E2 from BVDV (PDB entry 4ild, Li *et al.*, 2013 ▶). (*e*) The structure of the eukaryotic fusogen EEF1 (PDB entry 4oje, Pérez-Vargas *et al.*, 2014 ▶).

Class I fusogens are characterized by a seven-residue periodicity of nonpolar amino acids (called ‘heptad repeats’) that give rise to a central parallel trimeric α-helical coiled coil along the long axis of a rod-shaped molecule (Fig. 2 ▶; Igonet & Rey, 2012 ▶). The best characterized members of this class are the influenza virus HA (Wilson *et al.*, 1981 ▶; Bullough *et al.*, 1994 ▶) and the fusion protein (F) of paramyxoviruses (Yin *et al.*, 2005 ▶, 2006 ▶), but this class also includes fusion proteins from retroviruses, coronaviruses and filoviruses (see Baquero *et al.*, 2013 ▶ for a review). Both the pre-fusion and post-fusion structures of class I fusion glycoproteins are trimeric. In most of the cases, the subunits constituting the trimer result from the proteolytic cleavage of a precursor into two fragments. The resulting C-terminal fragment, which is anchored in the viral membrane by a hydrophobic transmembrane (TM) domain, bears a hydrophobic fusion peptide at or near its amino-terminal end that is buried at a protein–protein interface in the pre-fusion state (Fig. 2 ▶). This peptide gets inserted into the target membrane during fusion. Thus, in the post-fusion conformation, the shape of the trimeric molecule resembles an elongated hairpin-like structure with the fusion peptide and the TM domain located at the same end, as expected at the end of the fusion process. 

Until recently, class II proteins had only been found in flaviviruses (protein E) and alphaviruses (protein E1), which share many key characteristics (reviewed in Modis, 2014 ▶). The first X-ray structure of a class II glycoprotein was that of the tick-borne encephalitis (TBE) flavivirus E protein ectodomain (Rey *et al.*, 1995 ▶), solubilized from virions by limited trypsin digestion. Similar structures are now available for the ectodomain of denguevirus types 2 and 3. The polypeptide chain of the E protein is organized in three globular domains, essentially constituted by β-sheets (Fig. 2 ▶). The hydrophobic fusion loop in the virion is located at the tip of domain II, buried by interactions with domain III of the adjacent monomer in the EE dimer. These proteins are attached to the viral membrane *via* a C-terminal TM anchor, which is linked by a flexible ‘stem’ region to the ectodomain (Fig. 2 ▶). The envelope proteins from flaviviruses and alphaviruses assemble into icosahedral outer shells, but the mode of assembly differs in the two families, with alphaviruses forming canonical (*T* = 4) quasi-equivalent assemblies (Lescar *et al.*, 2001 ▶; Zhang *et al.*, 2011 ▶) and flaviviruses forming unusual non-equivalent icosahedral assemblies (Mukhopadhyay *et al.*, 2003 ▶; Zhang, Ge *et al.*, 2013 ▶. 

Recent data show that the class II fold is more widely distributed than previously anticipated. Indeed, the crystal structures of the glycoprotein C (Gc) from Rift Valley fever virus (RVFV) reveal a class II fusion protein fold (Dessau & Modis, 2013 ▶). RVFV belongs to the phlebovirus genus in the *Bunyaviridae* family, unrelated to flaviviruses or alphaviruses (Modis, 2014 ▶). The structure of RVFV Gc is strikingly similar to flavivirus E structures. In particular, both viruses share the same head-to-tail configuration of the protein dimers, with the fusion loop buried at the dimer interface (Fig. 2 ▶). Also, the two fusion loops have the same tightly folded glycine-rich structure suggesting that phleboviruses may be evolutionarily related to alphaviruses and flaviviruses.

In another recent advance, the E1 protein of rubella virus, which belongs to the same *Togaviridae* family as alphaviruses, was found to have a class II fold, although with a more divergent structure that shows important differences in the fusion loops (DuBois *et al.*, 2013 ▶). In addition, rubella E1 does not form icosahedral assemblies.

Very recently, two independent groups have solved the structure of the larger envelope protein, E2, from the pestivirus bovine viral diarrhea virus (BVDV) (Fig. 2 ▶), another member of the *Flaviviridae* family (Fig. 2 ▶). The structure unveiled that E2 has marked differences with the rest of the class II fusion proteins, defining a new structural class of fusogens (El Omari *et al.*, 2013 ▶; Li *et al.*, 2013 ▶).

Class III proteins are trimeric before and after fusion and share structural characteristics with both class I and class II fusion glycoproteins (Fig. 2 ▶). A class III fold was identified in the structures of the ectodomains of the fusion glycoproteins G of vesicular stomatitis virus (VSV) and B (gB) of herpes simplex virus 1 (HSV-1). Their comparison revealed an unanticipated structural similarity between the two proteins, although no sequence similarity had previously been detected. The structures of the Epstein–Barr Virus (EBV) gB and baculovirus gp64 fusogens also belong to this class (reviewed in Baquero *et al.*, 2013 ▶). VSV G is the only class III fusion protein for which the X-ray structures of both the pre-fusion (Roche *et al.*, 2007 ▶) and post-fusion (Roche *et al.*, 2006 ▶) states have been determined. The VSV G protein possesses both receptor-binding and fusion-promoter activities. As in the case of influenza virus, binding of rabdovirus G to a poorly characterized receptor at the cell surface induces endocytosis of the virus particle. Acidification of the endosome triggers G for membrane fusion. However, in contrast to all other fusion proteins, the low-pH inactivation of VSV G is reversible. The crystal structure of the G protein in the pre-fusion conformation revealed two fusion loops reminiscent of class II proteins that are oriented downwards towards the viral membrane. After low-pH exposure, the fusion domain moves upwards by flipping relative to the central core of the trimer to form an intermediate pre-hairpin structure. This is followed by the reversal of the molecule around the central rigid block formed by lengthening of the central helix in the grooves of the central core in an antiparallel manner (Fig. 2 ▶). This six-helix bundle has an obvious resemblance to those of the class I proteins. It is likely that the transition of VSV G from the pre-fusion to the post-fusion conformation involves disassembly of the trimer into the monomers and reassembly into trimers upon interaction of the fusion loops with the target membrane (Albertini *et al.*, 2012 ▶).

While vesicle fusion is required for a number of essential biological processes such as exocytosis and synaptic transmission, cell–cell fusion processes are crucial in development. In all cases, membrane fusion follows the same steps already described for virus–host interaction. In analogy with virus–cell fusion, vesicle and cell–cell fusion requires the formation of highly stable protein assemblies that provide the energy necessary to overcome the repulsive forces of membranes in close proximity. Also vesicle and cell–cell fusion, as in viral fusion, require higher-order multimerization of the fusion proteins. The main difference between virus–cell fusion and vesicle or cell–cell fusion is that in the former process the protein fusogen is present only in the viral membrane. In contrast, the proteins involved in vesicle fusion and cell–cell fusion are initially inserted in the two membranes predestined to fuse. Studies of intracellular fusion events have revealed two families of fusion proteins, the SNARE [soluble *N*-ethylmaleimide-sensitive factor (NSF) attachment protein receptors] (Südhof & Rothman, 2009 ▶) and the dynamin-like ‘atlastin’ GTPases (Bian *et al.*, 2011 ▶; Byrnes & Sondermann, 2011 ▶). In both cases, membrane merger results from trans-oligomerization of molecules anchored in the opposed membranes, followed by a conformational change that pulls the two membranes towards each other (reviewed in Moss *et al.*, 2011 ▶). In contrast to vesicle fusion, cell–cell fusion entails the same set of fusion proteins in the two membranes. For instance, the exceptional process of hypodermal cell fusion in *C. elegans* to form a large multi-nucleated syncytium of all skin cells is driven by the epithelial fusion failure 1 protein, EFF-1. Unlike SNAREs and viral fusogens, EFF-1 has been shown to be required in both cell membranes for fusion. Recently, the 2.6 Å resolution X-ray structure of the EFF-1 protein has been determined by Felix Rey and colleagues (Pérez-Vargas *et al.*, 2014 ▶). The EFF-1 trimer shows the same three-dimensional fold and quaternary conformation of post-fusion class II viral fusion proteins, although it lacks the nonpolar ‘fusion loop’, indicating that it does not insert into the target membrane and suggesting that membrane fusion driven by EFF-1 entails trans-trimerization. The authors further show that the blocking of EFF-1 trimerization interferes with the fusion reaction. The study also provides evidence that the monomeric form of EFF-1 is metastable and that trimerization is irreversible, matching the properties of the pre- and post-fusion forms, respectively, of the viral counterparts, demonstrating an evolutionary link with viral fusion proteins (Pérez-Vargas *et al.*, 2014 ▶).

## 4. Genome delivery in bacteriophages   

Bacteriophages show widely diverse structures and types of nucleic acid genomes; they have helical or icosahedral capsids and may not include a lipid envelope. Phages range in structural complexity from very small and simple non-enveloped icosahedral viruses (*e.g.* the ssDNA phage ϕX174) and long but simple helical viruses (filamentous phages), to large tailed viruses (*e.g.* the dsDNA phages T4 or ϕ29). 

**Figure 3:**
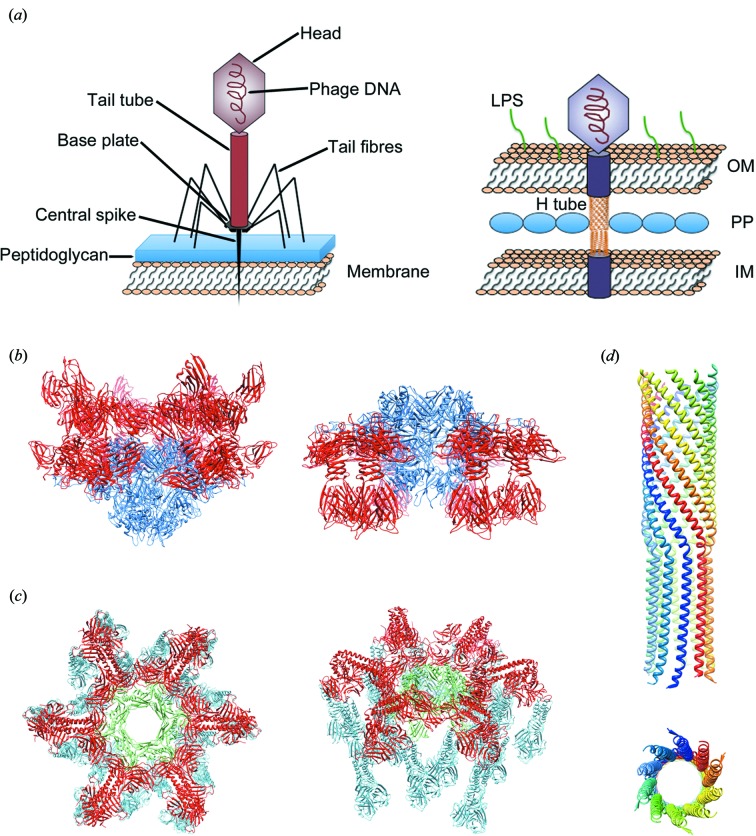
(*a*) Schematic representation of a tailed phage. LPS, lipopolysacharide; OM, outer membrane; PP, periplasmic space; IM, inner membrane. (*b*) The baseplate of phage P2 before activation, with the receptor-binding domains of the receptor-binding protein (red) pointing upwards, away from the bacterium (left; PDB entry 2wzp, Sciara *et al.*, 2010 ▶). The movement of 200° by the receptor-binding protein that completely reverses the orientation. The right panel shows the baseplate of P2 after activation by calcium ions with the receptor-binding domains pointing downwards, towards the bacterium (PDB entry 2x53, Sciara *et al.*, 2010 ▶). (*c*) The baseplate of phage TP901-1 (PDB entries 4div and 4diw, Veesler *et al.*, 2012 ▶), top view looking down the phage tail tube axis (left) and lateral view (right). (*d*) The structure of the H protein coiled-coil tube (PDB entry 4jpp, Sun *et al.*, 2014 ▶). Ribbon representation of the structure, with the monomers individually coloured. (bottom) Top view of the tube, with the N-terminus pointing to the viewer.

Bacteriophage tails are fascinating molecular machines specifically evolved to recognize host cells, penetrate the cell envelope barrier and deliver DNA into the cytoplasm (Fig. 3 ▶). The *Caudovirales* tails display very different sizes and morphologies with lengths ranging from ∼100 Å in some podophages to ∼8000 Å in some siphoviruses (reviewed in Fokine & Rossmann, 2013 ▶). Long tails of *Siphoviridae* and *Myoviridae* phages consist of the tail tip complex, which is responsible for host recognition and initiation of the infection process, the tail tube, which makes a conduit for genomic DNA, and terminator proteins, which terminate the tail assembly and create the binding interface for head attachment (Davidson *et al.*, 2012 ▶). The tail of *Myoviridae* phages also contains a contractile sheath surrounding the tail tube (Leiman *et al.*, 2010 ▶; Leiman & Shneider, 2012 ▶). The tail tip complex has different size and morphology in different phages. Phages which use protein receptors for cell binding usually have conical tail tips [*e.g.*, SPP1 or λ (Plisson *et al.*, 2007 ▶; Pell *et al.*, 2009 ▶)], whereas phages using polysaccharide receptors usually have elaborate baseplates at the distal end of the tail [*e.g.*, T4 or the lactococcal phages TP901-1 and p2 (Leiman *et al.*, 2010 ▶; Veesler *et al.*, 2012 ▶; Sciara *et al.*, 2010 ▶)]. Furthermore, phages usually have side-tail fibres or spikes, attached to the periphery of the tail tip complex, as well as a central tail spike (Fig. 3 ▶).

Bacteriophage baseplates vary in size and complexity. The most extensively studied *Myoviridae* baseplate is that of phage T4, which is composed of ∼140 polypeptide chains of at least 16 different proteins. The structures of T4 baseplate before and after tail contraction have been determined using cryo-electron microscopy (Kostyuchenko *et al.*, 2005 ▶; Leiman *et al.*, 2004 ▶) and the structures of nine baseplate proteins have been determined using X-ray crystallography (Leiman *et al.*, 2010 ▶). The T4 baseplate attaches six long tail fibres of ∼1450 Å length, which reversibly bind to the *Escherichia coli* lipo­polysaccharide (LPS) and/or OmpC molecules, serving for primary host recognition. It also has six short tail fibres attached to its periphery. Upon binding of the long tail fibres, a signal is transmitted to the baseplate causing the six short tail fibres to extend and bind irreversibly to the LPS, resulting in a conformational rearrangement where the baseplate switches from a dome-shaped form to a star-shaped conformation which, in turn, triggers the contraction of the tail sheath causing the specialized tip of the inner tail tube to puncture the outer membrane. The genome then passes through the tail tube into the cytoplasm (Leiman *et al.*, 2004 ▶; reviewed in Fokine & Rossmann, 2013 ▶).

Recent crystallographic and electron microscopy studies have revealed the structures of two *Siphoviridae* baseplates, those of phages p2 and TP901-1, infecting Gram-positive *Lactococcus lactis* (Sciara *et al.*, 2010 ▶; Veesler *et al.*, 2012 ▶). The baseplate of phage P2 is composed of three protein species. The central part of the baseplate is formed by a circular hexamer of ORF15 proteins with a central hole. A trimer of ORF16 is located at the bottom of the baseplate, forming a closed dome that does not allow DNA passage. Six ORF18 trimers are attached to the central ring, each trimer interacting with a carboxy-terminal extension of an ORF16 monomer. ORF18 is the receptor-binding protein (RBP) and electron microscopy reconstructions show that these proteins point upwards, towards the capsid in the free virion. In the presence of Ca^2+^, a cation essential for infection, the RBP complex is rotated 200° to point downwards towards the host cell. This conformational change of the baseplate also leads to the separation of three ORF16 monomers, opening up a hole in the centre of the baseplate, presumably allowing passage of DNA into the host (Fig. 3 ▶).

The baseplate of phage TP901-1 is composed of multiple copies of four different proteins. The centre is a hexameric circular core formed by the Dit protein. From the core, six arms emanate, each arm being composed of a trimer of the BppU protein. The arms are α-helical until the elbow. The rest of the arm points downwards and forms an adaptor domain for receptor-binding proteins. Three trimeric receptor-binding proteins bind to each adaptor leading to a total of 54 sites for binding the host cell envelope saccharides (Fig. 3 ▶). The hole in the centre of the hexameric core is filled by a central tail fibre (Tal) as seen in the electron microscopy reconstruction (Veesler *et al.*, 2012 ▶). The receptor-binding proteins point downwards, *i.e.* towards the host bacterium, ready for adhesion. No Ca^2+^ ions are necessary for activation of TP901-1, suggesting that conformational changes are probably not needed for receptor binding. How receptor binding is related to DNA transfer in these phages is less clear; perhaps the strong binding with up to 54 receptor molecules pushes the central tail fibre against the cell wall and the force exerted by the cell wall against the tail is sensed by the other end of the tail fibre, which opens a hole at the end of the tail tube.

Very recently, Rossmann and colleagues have demonstrated that the small single-stranded (ss)DNA bacteriophage ϕX174 possesses a mechanism of DNA translocation similar to that described for the tailed phages. However, the ϕX174 ‘tail’ protrudes from the virion only at the time of infection (Sun *et al.*, 2014 ▶). ϕX174 is a small icosahedral microvirus with a circular ss(DNA). X-ray and electron microscopy studies showed that the virus capsid has spikes on all pentameric vertices (McKenna *et al.*, 1992 ▶). The 260 Å-diameter capsid is constructed from 60 copies of the F protein. In addition, each of the 12 spikes consists of five G proteins, protruding 32 Å above the F-protein shell. The capsid also contained 60 copies of the DNA-binding protein J and ten to 12 copies of the DNA pilot protein H. Until now, the structure and location of the H proteins remained unknown as all structure determinations of this phage made use of the icosahedral symmetry. Rossmann and colleagues have now solved the crystal structure of the central domain of the H protein at 2.4 Å resolution. It consists of a 170 Å-long α-helical tube built from ten α-helices (Sun *et al.*, 2014 ▶). Each monomer is kinked at approximately the middle of the molecule, resulting in a tube with their amino termini arranged in a right-handed super-helical coiled coil and their carboxy termini arrayed in a left-handed super-helical coiled coil (Fig. 3 ▶). The N-terminal domain, which is slightly conical, has a minimum internal diameter of approximately 22 Å, whereas the internal diameter of the C-terminal domain is about 24 Å. With a minimum internal diameter of 22 Å, the H tube can easily accommodate two unpaired ssDNA strands with intercalated bases in a similar way to that in which the circular ssDNA genomes of filamentous bacteriophages are packaged into cylindrical fivefold symmetric capsids with comparable inner dimensions (Russel *et al.*, 2006 ▶). The H tube also appears long enough to span the periplasmic space, with both N- and C-terminal regions rich in Ala, Gly and Ser residues, which have high occurrence in transmembrane helices. Therefore, these regions would probably anchor the tube in the *E. coli* inner and outer membranes.

## 5. RNA-dependent RNA polymerases   

**Figure 4:**
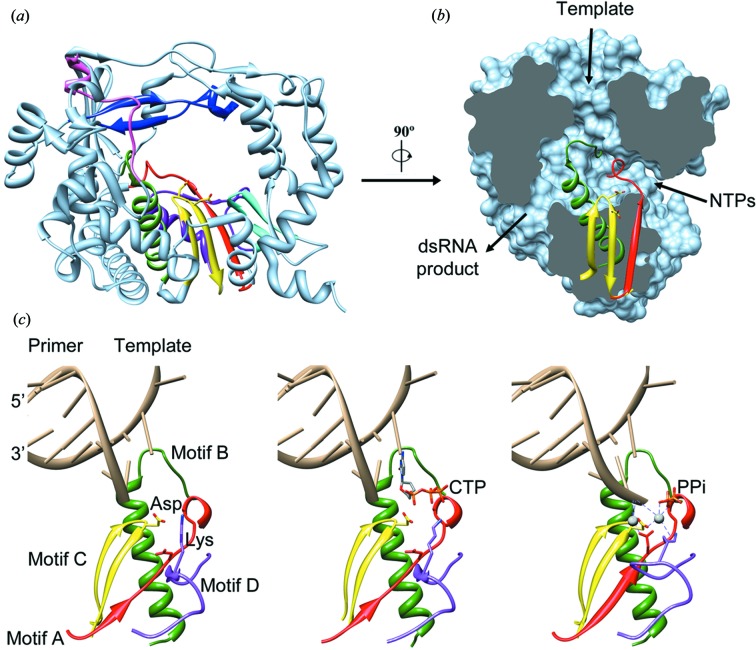
Overal structure of a viral RdRP. (*a*) Ribbon representation of a typical RdRP (FMDV 3D, PDB entry 1u09, Ferrer-Orta *et al.*, 2004 ▶). The seven conserved motifs are indicated in different colours: motif A, red; motif B, green; motif C, yellow; motif D, purple; motif E, cyan; motif F, blue; motif G, pink. The side chains of the catalytic Asp residues in the active site are also shown as sticks. (*b*) Lateral view of a surface representation of the enzyme (grey) that has been cut to expose the three channels that are the entry and exit sites of the different substrates and reaction products. The structural elements that support motifs A, B and C are also shown as ribbons. (*c*) Sequential structures illustrating the movement of the different residues within the palm domain from a binary RdRP-RNA open complex (left) to an RdRP-RNA-rNTP open ternary complex (middle), and a closed ternary complex (right). Image based on different poliovirus elongation complexes. The different structures correspond to the 3D-RNA (PDB entry 3ol6, Gong & Peersen, 2010 ▶), 3D-RNA-CTP open complex (PDB entry 3olb, Gong & Peersen, 2010 ▶) and 3D-RNA-CTP closed complex (PDB entry 3ol7, Gong & Peersen, 2010 ▶) structures of poliovirus elongation complexes, respectively.

RNA-dependent RNA polymerases (RdRPs) are the catalytic components of the RNA replication and transcription machineries and the central players in the life cycle of RNA viruses. RdRPs belong to the superfamily of template-directed nucleic acid polymerases, including DNA-dependent DNA polymerases (DdDP), DNA-dependent RNA polymerases and reverse transcriptases (RT). All theses enzymes share a cupped right-hand structure, including fingers, palm and thumb domains, and catalyse phosphodiester bond formation through a conserved two-metal-ion mechanism (Steitz, 1998 ▶). A structural feature unique to RdRPs is the ‘closed-hand’ conformation, as opposed to the ‘open-hand’ found in other polynucleotide polymerases. This closed-hand conformation is accomplished by interconnecting the fingers and thumb domains through the N-terminal portion of the protein and several loops protruding from fingers, named the fingertips, that completely encircle the active site of the enzyme (Ferrer-Orta *et al.*, 2009 ▶; Ng *et al.*, 2008 ▶). In the prototypic RdRPs, the closed ‘right-hand’ architecture encircles seven conserved structural motifs that are arranged in the order G, F, A, B, C, D and E containing a number of highly conserved amino acids (Fig. 4 ▶). The only exception to this scheme is found in Birnavirus and Permutatetravirus polymerases, where motif C is encoded upstream of motif A (Garriga *et al.*, 2007 ▶; Ferrero *et al.*, 2012 ▶). Each of the seven motifs in the RNA polymerase domain adopts a specific fold that extends beyond the regions of sequence similarity into the so-called homomorphs for most RNA virus RNA polymerases (Lang *et al.*, 2013 ▶). Three well defined channels have been identified in the RdRP structures, serving as: the entry path for template (template channel) and for nucleoside triphosphates (NTP channel) and the exit path for the dsRNA product (central channel) (Fig. 4 ▶).

The recent explosion of structural and functional information on viral RdRPs has provided insights into both initiation of RNA synthesis and the elongation process. Correct initiation of RNA synthesis is essential for the integrity of the viral genome. There are two main mechanisms by which viral replication can be initiated: primer-independent or *de novo*, and primer-dependent initiation, reviewed in van Dijk *et al.* (2004 ▶). Briefly, in the *de novo* synthesis, one initiation nucleotide provides the 3′-hydroxyl for the addition of the next nucleotide whereas the primer-dependent initiation requires the use of either an oligonucleotide or a protein primer as provider of the hydroxyl nucleophile. The members of the *Picornaviridae* and *Caliciviridae* families use exclusively the protein-primed mechanism of initiation. In this process, a tyrosine residue provides the hydroxyl group for the formation of a phosphodiester bond with the first nucleotide. Enzymes using primer-dependent initiation display a more accessible active-site cavity, enabling them to accommodate the small VPg protein that acts as a primer protein in RNA synthesis. The foot-and-mouth disease virus (FMDV) VPg protein lines the RNA-binding cleft of the corresponding RdRP (Ferrer-Orta, Arias, Agudo *et al.*, 2006 ▶), positioning its Tyr3 OH group as a molecular mimic of the free 3′-OH group of a nucleic acid primer at the active site for nucleotidylyl­ation, thereby initiating replication. In the presence of oligo­adenylate and UTP, the product of the reaction, VPg–UMP, can be observed in the crystal structure (Ferrer-Orta, Arias, Agudo *et al.*, 2006 ▶ ▶), at a position remarkably similar to the position of the template–primer RNA duplex (Ferrer-Orta *et al.*, 2004 ▶, 2007 ▶). After nucleotidylylation of VPg, some structural rearrangements of the RdRP follow, marking the transition from initiation to the elongation phase of RNA synthesis. By contrast, enzymes using the *de novo* initiation, Reovirus and Flavivirus RdRPs, contain additional structural elements that fill most of the active-site cavity, providing a support platform for the primer nucleotides, thereby enabling *de novo* initiation of RNA synthesis (Choi & Rossmann, 2009 ▶; Harrus *et al.*, 2010 ▶). Crucially, these protrusions are also able to undergo large conformational rearrangements to facilitate translocation of the RNA recently synthesized (Butcher *et al.*, 2001 ▶; Mosley *et al.*, 2012 ▶).

The replication elongation process can be roughly divided into three steps, including nucleotide selection, phospho­diester bond formation and translocation to the next nucleotide for the subsequent round of nucleotide addition. The structures of a large number of RdRP-RNA-rNTP replication–elongation complexes have been determined for different members of the *Caliciviridae* and *Picornaviridae* families. These studies have provided important insight into the structural alterations associated with each catalytic step (Ferrer-Orta *et al.*, 2009 ▶; Lescar & Canard, 2009 ▶; Ng *et al.*, 2008 ▶; Zamyatkin *et al.*, 2008 ▶, 2009 ▶; Gong & Peersen, 2010 ▶; Gong *et al.*, 2013 ▶; Garriga *et al.*, 2013 ▶; Sholders & Peersen, 2014 ▶). These structures indicated that RdRPs use subtle rearrangements within the palm domain to fully configure the active site for catalysis upon correct rNTP binding (Fig. 4 ▶). Among these movements, a flexible loop at the N-terminus of motif B assists in the correct positioning of the template nucleotide in the active site, facilitating the binding of the incoming rNTP *via* base pairing to a fully prepositioned templating base and stacking with the primer (Ferrer-Orta *et al.*, 2007 ▶, 2009 ▶; Garriga *et al.*, 2013 ▶). Binding of the correct nucleotide then induces the realignment of β-strands in the palm subdomain that includes structural motifs A and C, resulting in the repositioning of the catalytic aspartate in motif A to allow interactions with both the metal ions required for RdRP function (Zamyatkin *et al.*, 2008 ▶, 2009 ▶; Gong & Peersen, 2010 ▶; Gong *et al.*, 2013 ▶). Recent data also suggest that a conserved lysine residue within motif D can coordinate the export of the inorganic phosphate (PPi) group from the active site, once catalysis has taken place (Yang *et al.*, 2012 ▶), thereby triggering the end of the reaction cycle and allowing translocation of RNA. Finally, structural and functional data in enteroviruses indicate that steric clashes between the motif-B loop and the template RNA would also promote translocation (Sholders & Peersen, 2014 ▶).

Furthermore, very recently Ng and colleagues provided evidence for conformational changes occurring in the calicivirus RdRPs during catalysis. These authors compared the crystal structures of the RNA-dependent RNA polymerase from the human Norovirus (NV) determined in more than ten different crystal forms in the presence and absence of divalent metal cations, nucleoside triphosphates, inhibitors and primer-template duplex RNAs. These structural comparisons revealed that, in addition to the active-site closure, the NV RdRP exhibits two other key changes: a rotation of the central helix in the thumb domain by 22°, resulting in the formation of a binding pocket for the primer RNA strand, and the displace­ment of the C-terminal tail region away from the central active-site groove, which also allows the rotation of the thumb helix (Fig. 4 ▶).

Oligomerization of RdRPs has been reported for a number of Picornavirus, Flavivirus and Calicivirus enzymes. Oligomerization may lead to cooperative template binding, lattice formation, and a stimulation of viral RNA polymerase activity *in vitro* (Spagnolo *et al.*, 2010 ▶; Lyle *et al.*, 2002 ▶; Högbom *et al.*, 2009 ▶), which in Flavivirus appears to be specific for the initiation of RNA synthesis (Luo *et al.*, 2000 ▶). Also inactive RdRPs can induce stimulation of activity and participate in array formation, which has led to the hypothesis that this mechanism has evolved to stabilize the enzyme during replication. The dimerization of the Hepatitis C virus (HCV) RdRp has been proposed to be mediated by the thumb domain (Chinnaswamy *et al.*, 2010 ▶), whereas in PV, residues in the N-terminal part of the polymerase domain and two interface I aspartates appear to be crucially involved (Spagnolo *et al.*, 2010 ▶).

RdRPs synthesize RNA using an RNA template. This biochemical activity, not present in mammalian cells, offers the opportunity to identify very selective inhibitors of this viral enzyme. Antiviral drugs targeting the RdRPs may either directly inhibit polymerase activity or essential interactions with the RNA template, or RdRP–RdRP contacts promoting oligomerization, or interactions with other regulatory proteins. The detailed structural and mechanistic understanding of the conformational changes that occur during catalysis is essential not only for understanding viral replication at the molecular level but also for the design of novel inhibitors capable of trapping the enzyme in specific conformational states. The Flaviviruses, Hepatitis C virus, Dengue virus and West Nile virus, as well as calicivirus NV are clear illustrations of the extent of efforts directed towards developing drugs that inhibit viral replication (Powdrill *et al.*, 2010 ▶; Malet *et al.*, 2008 ▶; Gentile *et al.*, 2014 ▶; Caillet-Saguy *et al.*, 2014 ▶; Eltahla *et al.*, 2014 ▶).

## 6. Viral proteases   

In most single-stranded RNA viruses, some double-stranded RNA viruses and retroviruses with polycistronic genomes, various functional protein domains are initially expressed as a single polyprotein. Cleavage of the polyprotein into individual functional units is essential for their survival. Hence, polyprotein processing is an integral step in the replication of these viruses. Extensive structural studies on proteases of animal viruses have been carried out as they are potential candidates for antiviral therapy. As of July 2014, the PDB has 307 entries of viral proteases of which 276 are X-ray crystal structures. When filtered at 90% sequence uniqueness, the number of proteases is still as large as 93 of which 60 are structures with bound ligands. Viral proteases possess many unique features which distinguish them from cellular and bacterial proteases. These features include stringent specificity, regulation of activity, novel constellation of residues at the active site, amino-acid sequence unrelated to other proteases of the same class, and adaptability to multiple roles.

Viral proteases are very stringent in their substrate specificities, unlike cellular proteases whose specificities are normally dependent on the P1 residue only (Kay & Dunn, 1990 ▶). In many viral proteases, the efficiency of cleavage at a particular site of the polyprotein is dependent on the sequence on either side of the cleavable bond and P1 to P4 and P1′, P2′, *etc*. are also important for specificity. The conformation and accessibility of the peptide bond also contribute to the susceptibility to cleavage. Each susceptible site in a polyprotein may have a different cleavage profile. This allows a way of controlling the cleavage events such that the proportions of individual domains are controlled and their activities regulated (Babé & Craik, 1997 ▶; Spall *et al.*, 1997 ▶). The stringent substrate specificity of viral proteases has led to their application in molecular biology as a reagent for removal of affinity tags from fusion proteins. The most widely used viral protease for such applications is the tobacco etch virus NIa protease (Kapust & Waugh, 2000 ▶).

Many novel combinations of active-site residues have been identified among viral proteases. One such example is the serine-like cysteine protease family of viral proteases, which possesses a cysteine instead of a serine in the catalytic triad in a trypsin- or chymotrypsin-like fold (Bazan & Fletterick, 1988 ▶). The crystal structure of the HRV-14 3C^pro^ revealed the adjustments in the catalytic site required for accommodating a cysteine in the place of serine in the catalytic triad of serine-like cysteine proteases (Matthews *et al.*, 1994 ▶). The serine proteases of the cytomegalovirus family have a catalytic triad of histidine, histidine and serine (Qiu *et al.*, 1996 ▶). The members of the birnavirus family possess a protease with serine and lysine residues forming a catalytic dyad, similar to the Lon proteases in bacteria (Feldman *et al.*, 2006 ▶). The aspartic protease of HIV is also different compared to the cellular aspartic proteases in that it functions as an obligate dimer with the two catalytic aspartates contributed by two different subunits (Pearl & Taylor, 1987 ▶; Wlodawer *et al.*, 1989 ▶). A similar example of a composite active-site formation is the case of NS2^pro^ of HCV, which has a catalytic triad of histidine, glutamate from one monomer and cysteine from another (Lorenz *et al.*, 2006 ▶). The active-site geometry is similar to that in serine proteases. The existence of an altered catalytic triad might result in reduction of catalytic efficiency of viral proteases compared to cellular proteases. Such characteristics are suited to the viral life cycle since, instead of efficiency in proteolysis, viruses require greater stringency in specificity and regulation of function (Babé & Craik, 1997 ▶). In the cytomegalovirus protease with a novel catalytic triad, a slow proteolytic step may be optimal for viral assembly, as proteolysis should occur only after the capsid maturation, which may be a relatively slow process. Some of the viral proteases have a structural fold similar to serine proteases although there is no sequence similarity with cellular proteases. The Sesbania mosaic virus protease has a chymotrypsin-like fold and is shown to be similar to *Bacillus intermedius* glutamyl endopeptidase (Gayathri *et al.*, 2006 ▶). Athough the catalytic triad can be superposed well with serine proteases, the protease by itself is inactive (Satheshkumar *et al.*, 2005 ▶).

The catalytic function of viral proteases has been found to be regulated by other domains that may be attached covalently to the N- or C-terminus of the protease. These regulatory mechanisms also serve to control the time and place of proteolytic activity (Babé & Craik, 1997 ▶). It has been found that NS2A and NS4A act as cofactors for the regulation of activity of the NS3 proteases of dengue virus and hepatitis C virus, respectively. These cofactors also contain membrane-spanning domains, which might co-localize the proteases to the membrane before activation (Clum *et al.*, 1997 ▶). The oligomerization status of the viral proteases also appears to be variable compared to their cellular counterparts (Babé & Craik, 1997 ▶). The aspartyl protease of HIV is an obligate dimer. It has been demonstrated that the interaction of Sesbania mosaic virus (SeMV) protease with VPg in *cis*-(Protease-VPg) leads to the activation of the protease (Satheshkumar *et al.*, 2005 ▶). Similarly VPg covalently linked to the N-terminus of pepper vein banding virus (PVBV) protease NIaPro (VPg-NIaPro) results in the enhancement of activity (Mathur & Savithri, 2012 ▶). In both these cases, the interaction of the disordered VPg domain alters the structure and function of the interacting proteases. It was shown that the poliovirus 3CD precursor, which consists of the protease domain 3C^pro^ and the polymerase domain 3D^pol^, also exhibits protease activity similar to 3C^pro^, but the polymerase is active only as 3D^pol^ after the elimination of the 3C^pro^ domain (Harris *et al.*, 1992 ▶). Dengue virus protease shows 3000- to 6000-fold activation in the presence of the NS2B cofactor (Yusof *et al.*, 2000 ▶).

Another feature of viral proteases is their extreme sequence diversity, not only amongst themselves but also with respect to cellular proteases. No significant sequence identity has been found between viral proteases and cellular proteases, though the structural fold might be maintained (Bazan & Fletterick, 1988 ▶; Gorbalenya *et al.*, 1988 ▶). Most of the viral protease X-ray crystal structures have therefore been determined by *ab initio* methods of phase determination using isomorphous replacement or anomalous dispersion. In most cases, only the catalytic residues are conserved. The Coronaviral proteases contain an additional domain consisting mainly of α-helices at the C-terminus which is essential for dimerization (Anand *et al.*, 2002 ▶, 2003 ▶; Yang *et al.*, 2003 ▶). The high diversity of viral proteases might be due to the accelerated replication of the viruses and absence of a proof-reading mechanism in the replication of viruses with an RNA genome (Babé & Craik, 1997 ▶).

Many viral proteases perform more than one role. Hence, there are certain features in these proteases that confer unique sequence and structural properties compared to normal cellular proteases. The proteases of the alphaviruses have a compact structure resulting from shortening of loops such that their assembly into capsids is facilitated (Choi *et al.*, 1996 ▶). RNA-binding motifs incorporated into the canonical protease domains are observed with many viral proteases of RNA viruses (Mosimann *et al.*, 1997 ▶). In addition to the main function of polyprotein processing, viral proteases also appear to be involved in shutting down of host-cell translation by acting upon integral components of the host translation machinery. For example, the viral protease 2A^pro^ encoded by the HRV genome cleaves the eukaryotic initiation factor eIF-4G of the host, preventing host protein synthesis (Gradi *et al.*, 1998 ▶; Lloyd *et al.*, 1988 ▶). Sindbis core protein is not only a protease undergoing autocleavage from the polyprotein followed by self-inactivation, it also assembles to form the viral capsid (Hahn & Strauss, 1990 ▶). Helper component protease (HCpro) has been shown to act as a suppressor of post-transcriptional gene silencing (reviewed in Marathe *et al.*, 2000 ▶). However, the protease function may be independent of suppressor activity. Antiviral drug discovery with proteases of the animal viruses as targets will be benefited by consideration of the special features of these proteases.

## 7. Conclusions and future perspectives   

Since the first virus structures were determined at almost atomic resolution, about 35 years ago, virus X-ray crystallography has continued to provide an ever deeper and wider understanding of the virus world. However, many of the questions still remaining in virus structure and function will require an even bolder use of X-ray studies in combination with new experimental approaches, such as high-resolution electron microscopy and single-molecule techniques. Technological developments associated with synchrotron radiation with the advent of *in situ* diffraction and X-ray free-electron lasers, that will allow the possibility of single-virus-particle analysis, will undoubtedly influence our research on structural virology in the near future.
